# Evaluating Nursing Work Systems and Identifying Barriers for Robotic Technology Integration: Observational Study

**DOI:** 10.2196/89409

**Published:** 2026-06-01

**Authors:** Gina L Georgadarellis, Ellen Benjamin, Shannon C Roberts, Cidalia J Vital, Frank C Sup IV

**Affiliations:** 1 Mechanical and Industrial Engineering, Riccio College of Engineering, University of Massachusetts Amherst, Engineering Laboratory, 160 Governors Drive, Amherst, MA, United States, 1 413-545-2946; 2 Elaine Marieb Center for Nursing and Engineering Innovation, University of Massachusetts Amherst, Amherst, MA, United States; 3 Donna M and Robert J Manning College of Nursing and Health Sciences, University of Massachusetts Boston, Boston, MA, United States; 4 Baystate Health, Springfield, MA, United States; 5 Elaine Marieb College of Nursing, University of Massachusetts Amherst, Amherst, MA, United States

**Keywords:** technology, healthcare, robotics, nursing, human factors, ergonomics, workflow, hospital units, patient safety

## Abstract

**Background:**

Robotic technology has the potential to assist nurses, but the complexity and unpredictability of health care environments cannot be replicated in a laboratory setting. Furthermore, there is a lack of experiential evidence that robotic technology will meaningfully impact nursing. Collaborative development of technology and real-world usability studies offers the ability to address problems early in the design process when functional changes can be implemented.

**Objective:**

The purpose of this study was to use an observational study and systematically evaluate the work system of inpatient nurses to identify barriers to the integration of robotic technology. The objectives are to use an observational study of active hospital units to gain a deeper understanding of nursing tasks, workflow, and the health care setting; identify barriers to the integration of robotic technology using the people, environment, tools, and tasks (PETT) scan from the Systems Engineering Initiative for Patient Safety framework; and synthesize the work system components of the PETT scan into themes.

**Methods:**

We used the practice-oriented model of the Systems Engineering Initiative for Patient Safety, the PETT scan, to identify barriers for robotic technology use and innovation. A convenience sample of nursing staff was observed as they worked. Units included the emergency department, medical and surgical intensive care unit, preop or postanesthesia care unit, and general medical-surgical floor. The total number of observation hours per unit was based on data saturation, which occurred at variable times during the day shift, and was arranged with unit management. A total of 53 hours across 16 sessions were recorded. Multiple rounds of inductive and deductive coding were conducted. Briefly, a 3-phase iterative data analysis process was used—initial inductive content analysis, a deductive phase to organize emergent categories into a PETT scan, and finalization of the PETT scan with the identification of overarching themes.

**Results:**

Observations across all units yielded a broad set of barriers to integrating robotic and other health care technologies. Using the PETT scan, 78 barriers were identified and were summarized into 20 themes with supporting subthemes and exemplars.

**Conclusions:**

By systematically observing nursing workflows and synthesizing barriers into themes, this study provides new insight into the conditions that enable or constrain robotic integration. Findings suggest that robotic technologies are presently best suited for auxiliary and background roles. Broader integration into patient care workflows will depend on designs that align with clinical workflows, support interoperability and robustness, and address ethical, accountability, and coordination challenges inherent in nursing care, as well as maintained organizational support.

## Introduction

### Background

The health care system faces a rise in complex medical diagnoses, an increasing life expectancy of patients, and a critical shortage of nurses [1]. Nursing shortages are of particular concern, as inadequate staffing is a primary cause of missed and delayed nursing care [2]. Missed nursing care is associated with decreased nurse-reported care quality, increased length of patient hospital stay, higher patient mortality rates, decreased job satisfaction, and increased intent to leave the profession [3,4].

Efforts to combat missed nursing care and safe staffing are ongoing [5,6], but the complexity of real-world settings has limited the successful translation of research into policy and practice changes [7,8]. One potential opportunity to address nursing shortages and missed care is through the integration of robotic technologies into health care settings. Research suggests that nurses are receptive to the implementation of robotic technology to lessen their workload, improve nurse workflow, improve task prioritization, mitigate short staffing, and reduce burnout [9]. However, more work is needed to understand the barriers to successfully translating health care robotic integration in real-world practice.

The usage of robots in the health care setting is not novel; more than 3 decades have passed since medical robots were first developed to assist surgeons [8,10-12]. In fact, the first surgical robot cleared by the Food and Drug Administration appeared in 1993 with the Automated Endoscopic System for Optimal Positioning (Computer Motion Inc) [13]. While technically a class 2 laparoscopic device, the Automated Endoscopic System for Optimal Positioning allowed surgeons to control the orientation of a traditional laparoscope using foot pedals [14]. Since that time, robotic technology has expanded into many sectors, supporting a range of health care professionals. In nursing, automated dispensing cabinets (ADCs) are a prominent example of successful robotic integration into daily workflows. ADCs provide safeguards for nurses to remove the correct medications and have been shown to reduce errors, increase security, and track inventory in real-time [15,16]. Additionally, ADCs can increase work efficiency, offering a demonstrated reduction in the time spent on dispensing and preparing medications by an average of 32 minutes per 8-hour shift [17].

Mobile robots have also been shown to benefit nursing workflows by reducing medication dispensing and delivery times [18]. In a 2011 study, an academic medical center saw reductions in time from order to label printing, order preparation, and from medication checking to delivery when using the TUG Automated Robotic Delivery System (Aethon Inc) [19]. A decade later, another pilot study spanning 3 different academic and teaching facilities investigated the potential use of Moxi to support nursing and nonclinical staff (Diligent Robotics Inc). Moxi, classified as a delivery robot, is a mobile robot capable of tasks that include carrying patient supplies and delivering laboratory samples or medications [20]. Across 4 weeks, 1 site achieved a significant overall growth in average deliveries and a 100% compliance between patients’ fall-risk signage and their electronically documented fall-risk status [21]. Another pilot study found that medication delivery with Moxi was 4 times faster than anecdotal evidence provided by staff [22]. Quantitatively, Moxi had traveled 298 miles at the time of reporting, averaging more than 15,750 steps per day.

There is now a substantial body of research investigating the integration of robotic technology into nursing practice. A recent umbrella review found 13 reviews, representing 558 studies, exploring this topic [23]. Technology-supported automation offers several potential benefits, including support for data entry and information exchange, medication management and administration, patient data tracking and monitoring, infection control, and mobility or lift assistance [24]. Robotic technology is most promising when it supports tasks that are routine, repetitive, or high-risk [24]. Nurses themselves hold largely positive opinions of robotic assistance [23], perceiving a range of benefits that include improved efficiency, error reduction, reduced physical and cognitive workloads, and decreased burnout [9,24].

Despite its potential advantages, experiential evidence from real-world implementation of nursing robotic assistance remains limited, and few rigorous studies are exploring this topic [23]. This dearth of real-world integration may be due, in part, to limited nurse-specific collaborative robotic technologies. Most health care collaborative robots are designed for patients, not nurses, and despite their commercial availability in the United States, peer-reviewed scientific evidence on their usage and effectiveness is lacking [25].

Even where hospitals successfully adopt health care technologies, research evaluating their use and usability faces significant barriers, including difficulty recruiting participation by end users, time constraints, budgetary constraints, and ethical barriers [26]. Additional obstacles arise from the logistics of conducting on-site research, which often require establishing a research agreement between the hospital and the research institution, obtaining research personnel clearance involving background checks, up-to-date vaccination records, demonstrating human participants research ethics training, and obtaining approval from the institutional ethics board.

Nevertheless, research in naturalistic settings is necessary to deeply understand and advance health care technology. The complexity and unpredictability of health care environments are not easily replicated in laboratory settings. Collaborative technology development and real-world usability studies enable addressing problems early in the design process, when functional changes can be implemented, and benefits observed and documented. It is essential to work iteratively with nurses within the context of their work system and develop a richer understanding of authentic nursing workflows. Therefore, the purpose of this study was to systematically evaluate the work system of inpatient nurses to identify barriers to the integration of robotic technology.

### Study Definitions

The Food and Drug Administration does not provide a formal definition for the term “robot,” and there is still no universal or standard definition for robotic technology [27]. Additionally, the definition for medical devices is defined under Section 201(h) of the Food, Drug, and Cosmetic Act [28] is relatively broad and encompasses devices ranging in complexity from a bandage to a surgical robotic platform. Thus, it is important to explicitly define robotic technology in the context of health care to ensure understanding, especially among those who specialize in different disciplines or lack experiential exposure. The definitions used in this study are provided in Multimedia Appendix 1, and include medical device [28], robotic technology, robot [29], health care robot, autonomous technology [13,30], and sense-think-act paradigm (Multimedia Appendix 1).

### Systems Engineering Initiative for Patient Safety: A Practical Framework for Analyzing Work Systems

**Figure 1.**
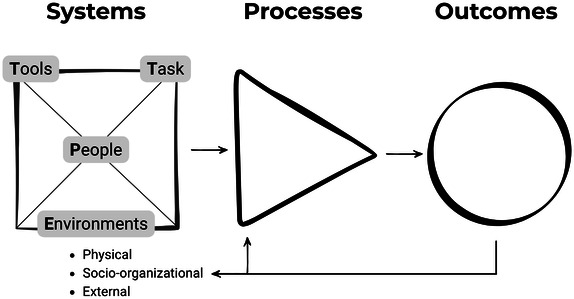
SEIPS 101 simplified model depicting work component feedback loop, adapted from Holden and Carayon. Work Systems is represented by a square and emphasizes the person as the center of the work system as well as interactions among the components; Processes is shaped as a triangle to convey the flow from left to right; and Outcomes is drawn as a circle, akin to the letter ‘O,’ to act as a visual reminder.

The Systems Engineering Initiative for Patient Safety (SEIPS) model integrates human factors and health care quality models [31], mainly balance theory [32], work system [33], and Donabedian’s [34] system-process-outcomes model of quality. SEIPS was created to address the challenge of applying human factors and systems engineering methodologies to improve patient safety research and the design of health care systems [35]. There have been different iterations of SEIPS since the model’s creation, and SEIPS has been implemented within nursing research [36]. SEIPS 101 provides a simplified practice-oriented model with the purpose of acting as a theoretical or practical framework to guide activity [37]. SEIPS 101 retains the 3 major work components (system, processes, and outcomes). The work system includes 4 work system elements—people, environment, tools, and tasks—which form the acronym “PETT.” The environment includes physical settings, socio-organizational conditions, and external context (Figure 1).

One tool from SEIPS 101 is the PETT scan, a matrix designed to ensure researchers consider the full breadth of the work system, specifically its people, environment, tools, and tasks [37]. Furthermore, traditional PETT scans include the interactions between work systems components. This tool may be used to indicate the aspects that hinder (barriers) or support (facilitators) people in the work system for each work system component or for component-component interactions. The PETT scan may be implemented in project planning or the conduct of research to systematically consider work system factors [37]. Therefore, the purpose of this study is to identify barriers to robotic technology integration within inpatient nursing work systems using a SEIPS analysis.

## Methods

### Overview

A qualitative observational study was implemented to elucidate the workflows of different units, their unique needs, key resources, and technologies used, and the barriers for robotic technology development or implementation. The original scope of the study was broad; researchers were interested in exploring opportunities for robotic technology usage and characterizing the current state of robotic technology use within acute care settings. Data were primarily collected between April 2023 and August 2023, with supplemental observational periods occurring until October 2024. We used the Standards for Reporting Qualitative Research (SRQR) reporting guideline [38] to draft this paper, and the SRQR reporting checklist [39] when editing (Checklist 1).

### Ethical Considerations

Approval for this research was obtained from the Institutional Review Board of the medical center (IRBNet 2008331). An information sheet describing the research study was distributed to unit management and was available at all times during the observation period. The study received a waiver of informed consent to minimize the burden on staff and interference with their work, and was determined to be exempt. To diminish the ability to identify staff, data collection used anonymized fieldnotes. Participants were identified using numbered pseudonyms corresponding to the job role they held at the time of observation (eg, nurse 1 and patient care assistant 1), and numbering was restarted at the start of each observation period. As data collection occurred while participants were working, participants were not compensated for this study.

### Setting

This study was conducted on multiple floors of a large, academic medical center in New England. This particular site was chosen due to the presence of multiple unit types, its strong interest in conducting translational research, and its history of successful previous collaborations with the researchers’ academic institution. To gather data on nursing workflows on a variety of units, observations were conducted in the emergency department (ED), medical and surgical intensive care units, preop or postanesthesia care unit (PACU), and a general medical-surgical floor.

### Participants

A convenience sample of nursing staff, facilitated by unit leadership, was selected from those working during the hours of data collection. The sample included direct care nurses, nurse leaders, patient care technicians, and charge nurses (Multimedia Appendix 2). Direct care staff were identified by their standardized dress attire (royal blue for the hospital units and by their badge for the PACU area). No staff identifiers were collected. Exclusion criteria included staff working with pediatric patients and patients with psychiatric disorders.

### Data Collection

Data were collected using overt participant observations. The focus of observation was nursing workflows and nurses’ interactions with and manipulations of their physical environment, including health care technology, medical devices, patient care supplies, and medications. Participants were asked brief questions and to think out loud or reflect upon their actions, decisions, strategies, and considerations in order to elucidate any tacit actions and to more deeply understand nurses’ behaviors and thought processes. The interactions with participants described above were limited, were not guided by a structured interview guide, aimed to minimize disruptions and distractions to staff work, and did not occur while the staff were working with patients. Additionally, when patient events or interruptions requiring privacy occurred, observation focus was diverted to capture unit details or events occurring in proximity. Unit charge nurses and/or nurse educators were asked questions regarding their thoughts on nursing workflow, decision-making related to technology use, and were asked to provide greater context for events that occurred during the observation. At the end of every observation period, field notes were digitally transcribed by the observer.

For each unit, the total number of observation hours was determined by data saturation (when the observed events and tasks became repetitive). Here, saturation was defined according to the standard of “informational redundancy,” when newly collected data does not yield anything new. Saturation was determined during data analysis, when no new barriers were identified, and codebooks were stable [40]. Periods of observation lasted approximately 4 hours each, with a minimum of 3 observation periods at each unit. Observations occurred at various times during the day shift, and scheduling was coordinated with unit management. Additional observations were scheduled based on findings during the initial observation hours.

Observations were conducted by a mechanical engineering researcher with a master’s-level education and previous research experience in nurse perceptions of robotic technology in health care and device usability (GLG). PhD-prepared nursing researchers with expertise in acute care and qualitative research (EB and CJV) provided consultation and ongoing support in protocol development, data collection, and data analysis.

### Data Analysis

Transcribed field notes were analyzed in NVivo 14 Plus software (Lumivero). A 3-phase iterative data analysis process of inductive and deductive content analysis guided by SEIPS work systems elements was used [41]. The use of both inductive and deductive analysis has been demonstrated in previous SEIPS research and combines the ability to identify emergent findings while applying validated theoretical frameworks [42].

As described above, the original scope of the study was broad, encompassing opportunities, barriers, and the current use of robotic technology by nurses in hospital settings. Data were first analyzed inductively, allowing codes and categories to emerge from the data. The purpose of this approach was to avoid bias from preconceived knowledge and not overlook potentially valuable insights. Descriptive codes were applied to text, and codes were categorized into emergent categories based on their similarities, differences, and patterns [41]. At this point in the analysis, it became apparent that there were few examples of current robotic use and that field notes demonstrated a much greater emphasis on barriers. The focus of analysis was revised to systematically characterize barriers to robotic usage. A second round of inductive analysis was performed with this new analytical focus.

Given the desire to comprehensively explore this phenomenon, the SEIPS model was applied to add robustness and ensure consideration of all work system elements. Codes and categories were analyzed and reorganized using deductive analysis to cluster findings according to the PETT framework. Finally, given the large number of identified barriers, thematic analysis was used to describe higher-order themes and patterns across barriers. In total, 3 investigators (GLG, EB, and CJV) conducted the content analysis, and a fourth investigator (FCS IV) also contributed to the final thematic analysis. Coding and category development were first completed independently by investigators, who then met regularly to discuss and refine analysis. Consensus was handled through discussions and iterative revisions until the team was satisfied.

### Trustworthiness

Efforts to increase trustworthiness were guided by Lincoln and Guba [43]. Strategies implemented included prolonged engagement with data, triangulation across multiple nursing roles and unit types, negative case analysis, and the creation of a rich audit trail using qualitative analysis software. Findings are supported by extensive field note evidence within the manuscript and appendices. The credibility of qualitative analysis was supported by multiple investigators who first coded independently and then reached a consensus. Investigators used memo-writing to increase reflexivity. The use of the SEIPS model added robustness to data analysis and coherence with previous health systems research. Finally, 2 engineering experts (SCR: human factors, FCS IV: mechatronics) were consulted to provide feedback on the PETT scan and to ensure the analysis was credible and well-aligned to a human factors and robotics perspective.

## Results

### Overview

Observations across 4 hospital units yielded a broad set of 78 barriers to integrating robotic and other health care technologies. Using the PETT scan framework, these barriers were categorized into 4 domains—people, environment, tools, and tasks (refer to “PETT scan” in Multimedia Appendix 3). For each of the barriers in the PETT scan, implications for robotic technology and an example field note are included. Table 1 summarizes these barriers by theme and supporting subthemes.

**Table 1.** Summary of the barriers identified using the people, environment, tools, and tasks scan framework from Systems Engineering Initiative for Patient Safety 101.

Work system element, barrier themes	Subthemes
People – patients	
Heterogeneity	Varying ages, sizes, levels of activity, and physical capabilities Varying levels of cognition and education Varying languages, cultures, and religions
Expectation differences	Patient expectations Nursing needs and expectations
Unpredictability	Noncompliance Negative patient behavior and aggression Misuse of technology Unpredictable clinical scenarios
People – staff	
Physical characteristics	Varying ages, heights, sizes, levels of activity, and physical capabilities Physically taxing equipment
Cognitive characteristics	Varying education, experience, training, dialects, and knowledge Bandwidth and emotional fatigue Inconsistent nomenclatures Insufficient training in technology Drug diversion or theft
Psychosocial characteristics	Varying motivations, needs, goals, culture, and religions Personal habits and preferences Fear of liability Reluctance to adopt new technology Satisfaction and frustrations with technology
Workflow considerations	Trade-offs between cost and speed or convenience Pacing mismatch between technology and workflow Shared ownership of supplies and equipment Failure to return or restock equipment Lack of lunch breaks and nutrition
Environment	
Cognitive burden of the environment	Dynamic and variable environments Overstimulation and distraction Alarm frequency Use of visual aids
Physical factors	Varying temperature and lighting Presence of liquid reagents and biohazardous materials Inconsistent unit layouts Dimensional constraints Inability to adapt infrastructure
Organizational culture and policy	Inconsistent policies and unit cultures Lack of support for nurse-led innovation Resistance to policy and procedure changes Regulatory compliance and oversight Inconsistent staffing roles Inadequate staffing levels
Accessibility	Trade-offs between accessibility and organization Inconvenient storage locations Considerations for patient visibility versus privacy
Technology or tools	
Physical considerations	Physical size and weight Need for transportability Need for high sensitivity and compatibility with personal protective equipment Need for mobility and concurrent use with other devices Screen visibility issues Impeding situational awareness
Speed or time considerations	Need for high speed Inconsistent network connectivity Data transfer and streaming time requirement
Maintenance or support	Need for robustness against physical damage and compatibility with diverse biological materials Inconsistent staff training and support; need for intuitiveness Technology and device maintenance demands staff, time, and money Frequency of update considerations
Device compatibility	Lack of backward compatibility and limited interoperability across manufacturers Incompatible devices should be visually distinct Lack of standardization across hospitals
Tasks	
Concurrent or competing tasks	Multitasking and interruptions Task redundancy Trade-offs between scheduled and unscheduled tasks
Task prioritization	Nursing prioritization and care left undone Technological bias of nursing judgment Need for patient-centric care requiring patient prioritization
Inconsistencies in task performance	Inconsistencies in staff performance Conflicting information Varying physical properties of materials Multimodal communication
Ethical considerations	Need for compassionate care Subjectivity in care Inconsistent staff roles and scopes Task shifting and delegation
Ergonomic considerations	Repetitive tasks Tacit and nuanced actions High dexterity User-friendly design

### People-Related Barriers: Patients

Patient-related barriers arise from patient heterogeneity, differing expectations between patients and staff, and unpredictability in patient behavior. First, patients are widely heterogeneous in their physical, cognitive, and psychosocial characteristics. Robotic technology must function across a wide range of patient sizes, strength levels, and cognitive abilities. For biomedical devices with sensors or that directly interface with patients, there is an even wider range of considerations for safety and the accuracy of biophysical data. In some care settings, such as the ED, technology must serve the full breadth of newborn, pediatric, adult, and geriatric patients.

*The nurse educator discusses available defibrillators. They had considered purchasing Stryker LifePak, but this only goes down to 2 joules and 1 joule is needed for pediatric patients.*

[Field Note June 30, 2023]

Technology must also serve patients with varying cultures, languages, and educational backgrounds. Similarly, patients carry a wide range of attitudes and expectations for medical technology. At times, these patient preferences were observed to conflict with nursing goals, safety, and workflow needs.

*Stat Nurse1 explains that the unit layout makes workflow difficult. The idea was to keep all operational equipment hidden from families…but from nursing’s perspective, the workflow safety is horrendous.*

[Field Note, July 25, 2023]

Finally, patients exhibit unpredictability in their behavior and decisions. Observations demonstrated several instances of patient noncompliance or an inability to adhere to nurses’ instructions. Equipment and technology must mitigate the risk of patient injury and harm and be resistant to theft, tampering, and patient interference. Technology must further contend with unpredictable decisions, such as patients acting illogically or changing their minds.

*A patient requested a walker and an escort from their room to the restroom. A patient care technician assisted the patient, and midway through their journey, the patient changed their request, wanting a wheelchair instead after potentially overestimating their mobility.*

[Field Note, June 09, 2023]

### People-Related Barriers: Staff

Similarly to patients, the heterogeneity of staff members creates barriers for technology use and implementation. Staff members encompass a range of ages, sizes, strengths, physical endurance, and mobility. Physical energy and emotional stamina may also vary between a single individual according to their shift, mood, and their ability to take breaks for food. The act of transferring a patient from their bed to a cat scan stretcher illustrates the physicality of patient care, involving a series of rolling, lifting, reaching, pulling, and pushing actions to move the patient and their equipment.

Staff heterogeneity also includes a range of educational levels, experience, technology training and knowledge, and mental capacities. Individual nurses demonstrate differing levels of focus, motivation, and ability to handle emotional challenges. One notable observation is the lack of consistency in staff nomenclature. Depending on their previous training and knowledge, staff may use different words to describe technology and medical supplies. Often, this nomenclature is inconsistent between staffing roles and with the language used in software systems. In practice, this can create confusion in care, impede supply management, and disrupt nurses’ ability to find supplies in stock rooms.

*The Nurse Educator works with supply management and wants to know what actually needs to be in the supply room. She has a list of everything in the supply room. The problem is that there are things in the room that they have no idea what they are…the barcode labels list items as RackBin using letters and numbers (eg, A1A1).*

[Field Note, November 20, 2023]

Just as medical technology should mitigate the risk of patient harm, it must also safeguard against harmful staff decisions. For example, the ADC that stores and dispenses medication is designed to reduce the risks of drug diversion and theft.

*Stat Nurse1 is having a discussion about narcotics and the scheduled drug check. This is a Friday chore at the Pyxis where certain drugs are checked…it takes about 20 minutes.*

[Field Note, May 19, 2023]

Additionally, staff demonstrate a range of preferences, expectations, and habits surrounding the use of technology. Nurses develop their own personal strategies and routines in care. These personal habits may create a reluctance to use technologies or adopt new devices. Across all units, observations revealed that nurses consistently use paper for notetaking rather than relying on electronic documentation. Daily frustrations with technology may also push nurses away from using technology and increase the use of nontechnical workarounds.

*Nurse1 explained that they were making a task list and going over all of their patients. The task list was on a plain piece of printer paper; paper folded in half and broken up into 6 sections. She wrote 1- or 2-word phrases with check box for each patient.*

[Field Note, April 28, 2023]

Finally, health care technology must be compatible with nursing workflows. Observations revealed many instances of poor fit between devices and nursing work, where technology caused significant delays or interruptions to patient care. When faced with obstacles, nurses may prioritize their own convenience or patient safety over considerations of cost or stewardship. Relatedly, staff members were frequently observed failing to restock or return supplies, leading to disorganization and exacerbating the challenge of finding them. One significant barrier is the lack of individual responsibility or accountability for health care technology. Resources are shared between staff members, with transient periods of “ownership” by individuals. This problem is even more pronounced with equipment or devices shared across nursing units.

*The ED nurse manager reports that cable management is a big issue. The department spent $23,000 tethering all the cables together to prevent nurses from taking cables, but this solution did not last long. After about 14 days, many had been cut by surgical shears. Nurse Educator1 states the issue is that nurses prioritize the patient even if it is at the expense of other nurses.*

[Field Note, October 09, 2024]

### Environment-Related Barriers

Environmental factors include the cognitive burden produced by the environment, physical factors, organizational culture and policy, and accessibility. First, environments are constantly changing and dynamic. Hospital units are full of distractions and stimulations, which can be exacerbated by health care technology. At one point in time, a nurse charting on a desktop computer may be confronted with a sounding bed alarm, the blinking red LED lights of a call bed, the audible keyboard strokes and mouse clicks, a rapid succession of beeps produced by the centralized monitoring system, and conversations between patients, visitors, and staff. Alarms, in particular, are highly prevalent and cognitively demanding. These alarms and notifications become barriers when staff struggle to distinguish between multiple competing sounds emanating from several devices and patient rooms. Furthermore, the volume and tone of alarms may not correlate to their urgency.

*An alarm goes off… [a few minutes later] the phone rings, another alarm for the pneumatic tube.*

[Field Note, July 25, 2023]

The environment has many physical properties that make technological innovation and usage difficult. Temperature varies across units, with the operating room notably cold and the emergency trauma room significantly hotter. Unit layouts are also inconsistent. Within the study site, one unit consisted of a long, narrow hallway with a centralized nursing station; one unit was broken into pods and did not have centralized supplies; one unit was U-shaped with resource-specific supplies; and another spanned 2 floors. All units demonstrated challenges with clutter and cramped spaces. Physical mess even included the presence of spills and liquid reagents on the floor, including biological and/or biohazardous materials. Light pollution from both in-room devices and the hallway was consistently present. In the PACU, each patient pod had a thin LED panel from floor to ceiling, emitting light, with access to privacy curtains that were sometimes used to dampen light. Underlying all these physical properties is the reality that, once built, physical layout is not easily altered or adapted to changing technologies or processes.

*PCA1 changed the trash; left the full trash bag on the floor in the doorway… Gloves were thrown in the trash at some point but missed trash; just lying on floor.*

[Field Note, May 19, 2023]

The hospital organizational context may also act as a barrier to technology innovation and usage. Established organizational policies make it difficult to change practices, and organizational cultures may not be conducive to nurse-led innovation. Ingenuity and change are also restricted by the need to adhere to external regulatory bodies. Nurses cited these external regulatory bodies, like the Joint Commission on Accreditation of Healthcare Organizations, as restricting their ability to implement innovative ideas and potential process improvements.

*A nurse comes in with a story of a missing hearing aid and issues with personal belongings again. They talk with the charge nurse. “Why can’t you just change things?” The response is, “because it’s a process.”*

[Field Note, July 06, 2023]

Finally, poor technology accessibility may compromise its usability and successful implementation. Frequently, technology was observed in areas that were not conducive to nursing workflows. Health care staff appeared to grapple with conflicting priorities, recognizing trade-offs between accessibility, speed of care, cleanliness and organization, unit aesthetics, and patient privacy or visibility.

*Stat Nurse1 needed to grab a stop cock/dead head for Patient2 and went into supply closet to look for it. It took Stat Nurse1 5-10mins total… [and] Stat Nurse1 didn’t even find what they were looking for; found something that used a stop cock and planned on taking it from that supply.*

[Field Note, May 19, 2023]

### Technology and Tool-Related Barriers

Technology and tool barriers included physical considerations, time and speed considerations, maintenance and support considerations, and device compatibility concerns. First, the scale of technology may pose challenges and become a barrier to use and innovation. While large technology takes up storage space and can impede movement, small technology may be hard to locate or be susceptible to theft. Because technology must be moved and transported throughout the hospital, barriers arise when device and equipment size impede portability. This is exacerbated by the fact that a single patient often requires multiple medical devices and equipment. Cables and wires, in particular, were observed causing significant issues, frequently forming tangles, restricting movement, and interfering with nursing care tasks.

*Nurse1 adjusts the bed for Patient2, changing the arm of the BP cuff and finger of O2 monitor. Wire management appeared a little tricky, as the lines got tangled.*

[Field Note, August 22, 2023]

The pace of technological change can also pose significant barriers to its successful integration. As described above, the pace of technological change was found to be impactful on nursing workflows. In addition to delays caused by improperly functioning technology, delays may arise from the inherent time required to transfer data between devices or from network connectivity issues. Wi-Fi connectivity appeared to be unit-dependent; in the ED, a nurse educator mentioned that the unit’s Wi-Fi was notoriously problematic due to the architecture and materials of the department. The unit has a “Downtime Cabinet” stocked with paper documentation (eg, rating scales; Situation, Background, Assessment, and Recommendation; discharge instructions; medication orders; and consents) for when nurses are unable to use their computers.

*Nurse Educator1 mentioned that some things don’t work, mentioned Wi-Fi issues specifically. The Wi-Fi doesn’t reach the corners.*

[Field Note, June 30, 2023]

Hospital technology robustness is also a significant barrier to technology use. Broken or malfunctioning equipment was a common occurrence observed to frequently impact nursing workflow. Often, these problems were not intuitive to fix, or the staff lacked sufficient training and support to correct them. Even when devices work as intended, technology still requires routine maintenance and updates, demanding staff time and expertise. Maintenance can become even more complicated when technology comes into contact with nonsterile biological and environmental materials.

*Nurse1 was on computer and needed to use handheld scanner, but scanner wasn’t acting properly, and she didn’t know why it wasn’t happy.*

[Field Note, May 30, 2023]

Finally, technology use may be compromised by device incompatibility. Health care technology is not standardized across different hospitals or even across hospital units. Therefore, a patient transferred from one location to another may have devices that do not easily integrate with a nurse’s workflow. In addition to inconvenience, safety concerns arise when incompatible technologies may appear compatible with inappropriate technologies because they are visually similar. This problem was highlighted by the hospital’s ED, which had recently acquired a new defibrillator. Following the implementation of this new device, several aspects of the unit’s workflow became infeasible. Previously purchased blood pressure sensors were not the same brand as the new defibrillator and therefore could not be used. The trauma room had a brand-specific monitor that worked only with the previous brand, but the unit did not intend to replace it due to the cost. The new device was also incompatible with the unit’s streaming monitors and other portable defibrillators. Although the cable physically fits in the port of other department devices, it did not function.

### Task-Related Barriers

Task factors include concurrent and competing tasks, task prioritization, inconsistencies in task performance, ethical considerations, and ergonomic considerations. Nurses often must manage multiple demands, engage in high levels of multitasking, and adapt to unpredictable events. Staff are also frequently interrupted and switch tasks quickly. Additionally, nurses may engage in redundant tasks, such as manually transcribing information from the computer onto a whiteboard or piece of paper. This highly dynamic and unpredictable workflow may not be reflected in technology’s task planning capabilities. In the ED, nurses rely on large paper to document care during highly critical situations because it better supports rapid documentation of multiple, simultaneous assessments, interventions, and narration.

*The ED has an initial assessment/intervention form… It gets scanned, but the scanning people don’t like it due to the large size of the paper. It’s 3 pages wide, 1 sheet that is trifolded.*

[Field Note, June 30, 2023]

Nurses manage these multiple, competing priorities by prioritizing tasks. Tasks are completed based on nurses’ assessments of priority, and many are left undone if they are not deemed important. The subjectivity and unpredictability of task prioritization may impede technology integration. Additionally, technology might shape or bias nursing work processes and influence the tasks that nurses deem to be most pressing.

*Preparing for a new admission and transfer… Usually, the charge nurse would look up the patient, but today the charge nurse had their own assignments. She talked on the phone very briefly while looking at the information and got a paper and copied information from the computer, noting that the ED didn’t give the patient medication so Nurse1 will have to do it.*

[Field Note, April 28, 2023]

The execution of nursing tasks is further complicated by inconsistencies in task performance. Tasks may be completed differently by different staff members depending on their role, knowledge, and understanding of current protocols. Tasks may also vary according to the other devices, tools, or medical supplies required. For instance, in observations, units offered multiple modalities for communication, including verbal, visual, phone, portable handheld devices, radio, and electronic communication. Or the task of administering a medication via an infusion pump may vary depending on the drug’s viscosity or fluid dynamics. In addition to protocols and procedures, when completing tasks, nurses often rely on their own clinical judgment and ethical considerations. Nursing care and task completion, therefore, can be highly subjective, variable, and even involve actions outside of nurses’ traditional role and scope.

*Nurse1 walked back to Patient1 and then walked with a patient’s family member to escort them out of the unit. She said that they don’t do that too often, will have a PCT or another worker do it if they’re able to, but will also give oral directions.*

[Field Note, May 30, 2023]

Finally, technology use and implementation may be compromised by the ergonomic demands of completing tasks. Many dexterous tasks occur and involve more than one hand. Nursing tasks can be repetitive, tacit, and nuanced, placing a high burden on nursing operators. This was showcased in the PACU with intravenous starts, a routine task. Intravenous starts required many bimanual and minute actions.

*Don gloves; apply tourniquet; assemble syringe and needle; open gauze; open swab; swab patient; check patient anatomy; air dry swab area by waving hands; insert needle; remove tourniquet.*

[Field Note, August 31, 2023]

## Discussion

### Principal Findings

Understanding the antecedents of robotic technology use in health care elucidates both health and nonhealth-related consequences [44]. Merging these barriers with design guidelines [45], robotic systems design processes [46], safety taxonomies [47], and standards [48] can provide technology designers with valuable insights for creating robotic technology that effectively supports and aligns with nursing work. The contribution of this study is a comprehensive and empirically based overview of the potential barriers to integrating robotic and digital technologies into inpatient nursing workflows, categorized by the SEIPS elements of work systems (Table 1). The implications of each of these barriers are detailed in Multimedia Appendix 3.

Nursing has been described as a complex adaptive system, characterized by unpredictability, dynamicity, adaptability, and nonlinearity [49]. Nursing workflows are often inconsistent, vary in pace, do not follow a linear sequence, and frequently rely on workarounds. Scholars have used the term turbulent to capture the variability and fragmentation of nursing workflows, which are further complicated by time pressures, multiple competing demands, and resource constraints that require multitasking and task shifting [50,51]. In addition to nonlinear work processes, nurses must adapt to changing patient behaviors, team composition, and unpredictable clinical events. This work occurs in a physical environment that is often cluttered, restricted, disorganized, noisy, and cognitively demanding. Together, the characteristics of nursing work present significant challenges to the design and implementation of robotic technologies, which often depend on linear, repeatable, and well-parameterized tasks. Robotic design, therefore, cannot generalize from controlled demonstrations but must model the boundaries of variability that nurses manage in real time. In reality, technology may be disrupted during usage and required to deviate from routine behavior during both scheduled and unanticipated events. Workflow must be investigated before implementation to ensure appropriateness and to facilitate integration. Future research should focus on capturing this variability and encoding appropriate responses across combinations of factors.

The realities of hospital systems add additional design complexities and constraints. Inpatient care settings comprise a multitude of independent and disparate people, including frontline staff, providers, administrators, patients, and families. These individuals represent a wide range of roles, expectations, psychosocial preferences, physical statures, and cognitive abilities. They are organized across multiple groups and departments, each representing their own culture, norms, rules, processes, and priorities. Health care systems have been critiqued for their disciplinary silos or “worlds,” illustrating the significant discontinuity between caregivers, community members, and administrators [52,53]. In addition to internal agents and stakeholders, hospitals manage the requirements of laws and external regulations. Collectively, this heterogeneity and organizational complexity limit the effectiveness of standardized approaches, force trade-offs between overlapping design options, and limit the ability to support multiple end users.

When designing robotic technology, adjustable hardware, multimodal interfaces, and flexible workflows can help ensure usability across a range of users and use cases. However, the heterogeneity of people, roles, and culture makes generalization difficult. Technology that may be positively received in one unit may not be reciprocated in other units, even within the same organization. The same applies to people, where people of different or even within the same role may not agree on the functions and features of technology. If this variability cannot be accounted for, the scope of scenarios to which technology can be applied must be explicitly defined. Furthermore, organizational complexity influences the feasibility of innovative technology, with implementation often depending less on clinical need than on existing spatial, financial, and policy constraints. Because innovation requires a significant upfront investment and the rigid funding structures many organizations operate under, it is difficult for decision-makers to ignore the cost of technology compared with its potential benefit or value to patient outcomes [54]. Furthermore, nurse-led ideas for technology usage and innovation need organizational support to be implemented, which may require organizational policy changes to allow nurse involvement in technology design during working hours [55]. Thus, designing robotic technology with organizational involvement is critical.

Observations of real-world interactions between nurses and existing devices underscored the fragility and inadequacy of health care technology. In practice, the use of health care technology was frequently impeded by incompatible devices, missing supplies and accessories, transportability barriers, malfunctions, breakdowns, and profound misalignments between device operation and nursing workflows. Usability was further constrained by suboptimal interfaces, poor screen visibility, and inconsistent hospital practices. Previous literature corroborates the significant demands placed on nurses to locate, manage, organize, transport, use, and maintain technology [56,57]. These misalignments between existing devices and nursing work have undermined nurses’ trust in technological solutions and added to frustration, as some technologies are perceived to increase nursing workload and complexity [58,59]. Tools that limit autonomy, impose a physical burden, increase cognitive workload, or interfere with empathy-driven or judgment-based activities may further undermine professional confidence and acceptance of robotics.

Overall, given the state of current robotic technology and the realities of nursing work systems, there appear to be particular challenges implementing robotics in roles that constrain the pace of time-sensitive nursing tasks, require real-time adaptability, directly interface with a diverse patient population, or risk disrupting trust in nurse-patient relationships. Combined with previously identified drawbacks of automation in nursing, where concerns include dehumanization of care, risk of deskilling, and equitable access [24], it is essential that future use of robotics in nursing does not impede the work system. The barriers identified in this study emphasize that people within the work system are heterogeneous and variable; the environment imposes physical and organizational limitations; technology and tools are often fragile and inadequate; and the tasks nurses perform are constrained by workflow, ethics, and ergonomics. Robotic systems must be designed deliberately, and to navigate these barriers, innovation will need considerable organizational support, time, and iterative design. We conclude that integration of present-day robotic technologies is most feasible in auxiliary and physically structured components of nursing work, as core bedside activities depend on frequent interruptions, shifting priorities, spatial variability, and distributed responsibility across staff, factors that robots cannot yet sense or interpret reliably enough to support effective clinical teaming. Acceptance will depend on whether robotic systems simplify rather than complicate care delivery. The most promising opportunities for robotic integration, therefore, lie in augmenting background processes, while the most critical limitations arise when systems compete with the adaptive and relational aspects of nursing work. Successful implementation will require designs that acknowledge and support the relational dimensions of nursing practice, while providing clear accountability structures and safeguarding nurses’ professional judgment.

### Limitations

As with many observational studies, there may be the occurrence of an observer effect where observing a phenomenon may change it [60]. Thus, participants may have altered their behavior due to the presence of an external observer. Additionally, because data collection was limited to what was visible or audible to the observer [61], and because the observer does not have a health care background, factors that influenced the participants’ decision-making processes may not have been sufficiently captured. This was mitigated by encouraging participants to think out loud, asking clarifying questions, and by including health care professionals in the data analysis stage.

Additional limitations arise from both the participants and the observer in this study. Because recruitment was facilitated by the unit’s leadership team, there is potential for selection bias, in which participants may have been chosen based on specific characteristics or workflow. Furthermore, the same participants may have been observed multiple times. In bedside nursing, each shift is different, and the work system component interaction varies even when the same individual is being observed. One method to mitigate both selection bias and repeat participants was to allow multiple staff members to be shadowed during each observation period, observe interactions with other health care members, and include at least 3 different observation periods per unit. Finally, while patient-related barriers were observed and discussed, this study did not include patients’ perspectives, and thus, the impact of technology on patients remains unexplored. Future work would benefit from including patients to elucidate how barriers affect those receiving care.

This study was conducted only during the day shift and excluded both the pediatric population and the population with psychiatric disorders, which may limit the generalizability of these findings. While 4 different units were included in this study to capture a broad range of nursing workflows, these findings may not be applicable to units that care for excluded populations or have a large night-shift population. Furthermore, because the purpose of this study was to identify barriers to the integration of robotic technology, one limitation is that opportunities for robotic technology are not explored extensively, and thus, the translational aspect of these findings is limited. The identification of barriers is essential, as realistic opportunities and design implementation requirements will be shaped by their presence. Exploring the connections among barriers, design requirements, and robotics-assisted solutions is an essential next step for future studies.

### Conclusion

By systematically observing nursing workflows and synthesizing barriers into themes, this study provides new insights into the conditions that enable or constrain the integration of robotics into clinical practice. The findings emphasize that successful implementation depends less on technical capability and more on adaptability within complex sociotechnical environments. Robotic design cannot generalize from controlled demonstrations but must model the boundaries of variability that nurses manage in real time. Present-day robotic technology is best suited to support auxiliary and background tasks that relieve, rather than replace, human effort; however, substantial opportunities remain to expand their role through human-centered designs that address workflow variability, coordination demands, and distributed accountability in nursing care.

Design must prioritize adaptability, workflow alignment, reliability, robustness, interoperability, and ethical clarity. Successful implementation will also require organizational structures that promote training, maintenance, and relational sensitivity. Viewing technologies as sociotechnical systems embedded in complex work environments, rather than stand-alone devices, can offer a pathway toward safer, more resilient human-centered automation. The PETT scan proved valuable in mapping these challenges and can serve as a practical framework for anticipating barriers in future translational research.

## Supplementary material

10.2196/89409 Multimedia Appendix 1 Definitions used in this study.

10.2196/89409 Multimedia Appendix 2 Participant job role definitions.

10.2196/89409 Multimedia Appendix 3 People, Environment, Tools, and Tasks (PETT) Scan barriers.

10.2196/89409 Checklist 1 SRQR reporting checklist.

## Abbreviations

ADC: automated dispensing cabinet
ED: emergency department
PACU: preop or postanesthesia care unit
PETT: People, Environment, Tools, and Tasks
SEIPS: Systems Engineering Initiative for Patient Safety
SRQR: Standards for Reporting Qualitative Research
