# Supplementary material for: Evaluating Nursing Work Systems and Identifying Barriers for Robotic Technology Integration: Observational Study
Source: J Med Internet Res. 2026 Jun 1;28:e89409. doi: 10.2196/89409 (PMC13225718; doi:10.2196/89409)
Supplement: Multimedia Appendix 1 [file jmir-v28-e89409-s001.docx]

This is a Multimedia Appendix to a full manuscript published in the J Med Internet Res. For full copyright and citation information see <http://dx.doi.org/10.2196/jmir.89409>

| Definitions used in this study | |
| --- | --- |
| Term | Definition |
| Medical device | A product (e.g., apparatus, machine, implant, in vitro reagent) that does not achieve its purpose via chemical action or metabolization and is intended to affect the structure or function of living beings for usage in the diagnosis, cure, mitigation, treatment, or prevention of diseases and conditions [1]. |
| Robotic technology | Devices and platforms that utilize robots |
| Robot | Machines that operate using the sense-think-act paradigm [2], with some level of autonomous functionality. |
| Healthcare robot | Robots used by professionals in the healthcare/clinical setting and perform tasks that interact in some capacity with patients, nurses, doctors, and other healthcare professionals |
| Autonomous technology | Technology that demonstrates the ability to deal with the environment and perform behaviors or tasks without human intervention for an extended period. There are different levels of autonomy, and 5 levels have been identified for surgical robots [3,4]:   - Level 0 No autonomy: Human performs all tasks manually. - Level 1 Robot assistance: Human has continuous control. Robot provides guidance and passive support - Level 2 Task autonomy: Human has discrete control, monitors, and intervenes. Robot performs certain operator-initiated tasks - Level 3 Conditional autonomy: Human selects task plan and strategies. Robot performs task with close oversight - Level 4 High autonomy: Human supervises. Robot makes independent decisions and performs task - Level 5 Full autonomy (no human needed): No human needed. Robot performs task independently. This is a fictitious “robotic nurse” that is capable of all procedures performed by a general nurse |
| Sense-think-act paradigm | A mental model to characterize how a robot operates. Robots use sensors to collect data from the environment, such as audio, pressure, temperature, light, and distance (sense), make decisions using algorithms based on the information collected (think), and perform actions in the real world by turning the information collected into instructions for behaviors (act) |

**References**

1. U.S. Food & Drug Administration. How to determine if your product is a medical device. 2022. https://www.fda.gov/medical-devices/classify-your-medical-device/how-determine-if-your-product-medical-device [accessed January 25, 2024].

2. Bekey GA. On autonomous robots. Knowl Eng Rev. 1998;13(2):143-146. doi:10.1017/S0269888998002033

3. Lee A, Baker TS, Bederson JB, Rapoport BI. Levels of autonomy in FDA-cleared surgical robots: a systematic review. Npj Digit Med. 2024;7:103. doi:10.1038/s41746-024-01102-y

4. Yang GZ, Cambias J, Cleary K, et al. Medical robotics—regulatory, ethical, and legal considerations for increasing levels of autonomy. Sci Robot. 2017;2(4):eaam8638. doi:10.1126/SCIROBOTICS.AAM8638
