# Supplementary material for: Evaluating Nursing Work Systems and Identifying Barriers for Robotic Technology Integration: Observational Study
Source: J Med Internet Res. 2026 Jun 1;28:e89409. doi: 10.2196/89409 (PMC13225718; doi:10.2196/89409)
Supplement: Multimedia Appendix 2 [file jmir-v28-e89409-s002.docx]

This is a Multimedia Appendix to a full manuscript published in the J Med Internet Res. For full copyright and citation information see <http://dx.doi.org/10.2196/jmir.89409>

| **Participant job role definitions** | |
| --- | --- |
| Role (abbreviation) | Definition |
| Direct care nurse (RN) | Registered nurses who have passed the Board of Registration in Nursing Examination and are licensed in the state where the academic medical center operates. Depending on the unit, may be required to have certain certifications and education levels. Perform and support a variety of care activities for patients as well as clinical tasks at the bedside. |
| Nurse leader (NE) | Registered nurses who perform management and supervision tasks for a unit. Includes roles such as:   - Nurse educators: Responsible for the planning, coordination, implementation, and evaluation of educational programs and activities that support newly licensed registered nurses transitioning into clinical practice, as well as nursing students. Focuses on fostering clinical competence, professional development, and role socialization through evidence-based education strategies - Nurse managers: Assumes accountability and responsibility for effectively executing philosophy, objectives, policies, and procedures of the service. Responsibilities are centered around: clinical practice and quality outcomes, human resource leadership, management of the environment, financial growth and stewardship, and strategic planning. |
| Charge nurse (ChN) | Registered nurses who are assuming the role as charge (shift supervisor) for the observation period. Act as a liaison between staff and management. Oversee the unit and the direct care nurses on shift. Responsible for scheduling, answering questions, ensuring patient quality of care, and sometimes will have their own patient assignments. |
| Patient care technician (PCT)* | Healthcare team members who act under the supervision of an RN. Assists with direct patient care (e.g., hygiene, mobility, vital sign monitoring) and performs a range of clinical tasks (e.g., phlebotomy, EKGs, specimen collection, Point of Care testing). This role also includes assisting with procedures, patient transport, equipment management, and supporting emergency response protocols. |

**PCTs were auxiliary participants. While not direct participants, observation notes include PCTs only when interacting with the nurse participant.*
