# Supplementary material for: Evaluating Nursing Work Systems and Identifying Barriers for Robotic Technology Integration: Observational Study
Source: J Med Internet Res. 2026 Jun 1;28:e89409. doi: 10.2196/89409 (PMC13225718; doi:10.2196/89409)
Supplement: Multimedia Appendix 3 [file jmir-v28-e89409-s003.doc]

This is a Multimedia Appendix to a full manuscript published in the J Med Internet Res. For full copyright and citation information see http://dx.doi.org/10.2196/jmir.89409

| **People, Environment, Tools, and Tasks (PETT) Scan barriers** | | | |
| --- | --- | --- | --- |
| Work System Factor | Barrier | Implication | Field Note |
| People (Patients) | Patient characteristics vary, including ages, sizes, levels of activity, physical capabilities, and levels of cognition. | Technology must function for patients with varying individual characteristics. For example, patients may inappropriately interact with technology, may not have the cognitive ability to understand technology, or may not have the physical ability required for usage. The hospital environment can often be stressful for patients and their behavior and capacity for understanding what is happening around them may be altered. | In the medical-surgical unit, a patient was observed interfering with one of the medical devices in their room, causing a “staff emergency” false alarm. In contrast, in the ICU, the patient was nonreactive and did not interfere with any of the multiple devices required for their care. In other units, nurses were observed making accommodations for patients, including using pediatric EKG for an adult patient with skin sensitivities to minimize the negative dermatological effects, or using a soft call bell with increased sensitivity to improve usability for a patient with dexterity issues. In the ED, where both adult and pediatric populations are treated, the FDA approved defibrillator options were limited due to the required range needed to serve both populations. |
|  | Patients’ preferences and expectations differ. Within the same patient, these preferences and expectations are variable. | Technology must be acceptable to patients with differing preferences and expectations. Conflicting views and situations may arise; thus, generalizability is difficult. | In the PACU, a patient had requested a walker. A PCT was assisting the patient and midway through their journey from their bed to the bathroom, the patient changed their mind and wanted a wheelchair. |
|  | Patients and staff have differing expectations of technology. | Staff (end-user) and patient (receiver) may not agree on functions and features of technology, making generalizability difficult. Explicit justification of design metrics chosen for prioritization is needed if staff usability conflicts with patient experience. Usability testing and interviewing is important to find balance. | In the ICU, a nurse commented on how the surgical layout was designed to keep equipment out of view from families. While beneficial for nonprofessionals, this tradeoff was made at the expense of the nursing workflow and safety. |
|  | Patients may speak different languages. | Technology must be usable for people who speak different languages. This may require translational services for verbal and written instructions and documentation. Using standardized, universal images and icons may mitigate the need for such services for the visually abled. | In the PACU, the portable call device did not function properly for a nurse speaking in a non-Northern American English dialect – when they said a name, the wrong person was called. When a different nurse speaking with a regional dialect used the same device, it worked as intended. In the medical-surgical unit, a non-English speaking patient was observed trying to communicate unsuccessfully. Three workers were observed trying to communicate with the patient at the same time. |
|  | Negative patient behavior occurs. | Patient behavior (e.g. noncompliance, aggression) impact technology usefulness and appropriateness. Additionally, safety of staff and patients may be impacted if technology is misused and/or damaged during such behavior. | In the medical-surgical unit, a patient with a lower limb amputation repeatedly tried to leave their bed, triggering the bed alarm and requiring nurse intervention each time. In the PACU, a patient was explaining to a healthcare provider that they do not like wearing their CPAP mask due to having an inadequate fit. The patient stated that overtightening the mask causes indentations on the skin. Conversely, securing the mask loosely causes sealing issues during movement. |
|  | Wide variety of scenarios may occur. | Technology must account for variable patient questions and education. Generalizability is difficult and challenges arise for autonomous systems due to this variability. If this variability cannot be accounted for, the scope of scenarios that technology can be applied to must be explicitly defined. Policies must be in place to define this role, and ethical considerations occur if this role is overstepped. Technology designers should account for operation outside of intended use, take actions that minimizes risks, and explicitly state consequences of misuse. | In the PACU, a patient asked for ice water multiple times. Their nurse had to explain that they could not have water before their procedure due to the potential need for intubation and associated risk of pneumonia. Other questions did not receive answers, such as when a patient asked a question about returning to work the next day. Their nurse remarked how the question was more suitable for their doctor. |
| People (Healthcare staff) | Staff use different words/nomenclatures to describe technology and medical supplies. | Confusion arises from staff who use different words/nomenclature to describe technology. Generalizability is difficult and large amounts of time and data may be needed to identify nomenclature synonyms. Standardization of terminology or the ability to customize labels and information systems to fit the unit’s nomenclature is needed. | In the PACU, a nurse educator was interested in elucidating if their supply closet is stocked with the appropriate medical supplies. They stated one problem they are facing is that the nomenclature of supplies is inconsistent, and nurses would not be able to know what they had in stock when reading the list of supplies provided by supply management. |
|  | Staff characteristics vary, including ages, sizes, levels of activity, physical capabilities, and levels of education. | Technology must function for staff with varying individual characteristics. | In the medical surgical unit, equipment such as workstation on wheels, tablet stands, IV poles, and chairs, was often an obstacle in the hallway that decreased bed maneuverability. In the ICU, transferring a patient from the bed onto the CT patient table required 5 people. The patient was on a green transfer cloth and staff used a plastic transfer board to assist the task. Working in groups, one side rolled the patient towards them while the other side placed the transfer board under the patient. Then all five staff members worked in unison to push/pull the patient onto the patient bed of the CT machine. |
|  | Inadequate staffing and retention impede knowledge sharing and experience. Hospitals have limited resources to support training and on-going education of staff. Limited time for training and process improvement. | Device documentation and training design and additional support documentation are important. Non-expert users should be able to navigate documentation easily and quickly. Intuitive usage and design are important as staff may not be properly trained to use the device and may be relying on their internal mental model or prior experience with similar devices, or the manual may not be accessible in a timely manner. The inclusion of device instructions and/or pictorial representations directly on the device may be helpful as to allow usage without the presence of manuals. | In the ED, a nurse was contemplating factors that slow down their workflow and specifically mentioned an issue with older nurses leaving. In a different instance, a PCT noted that there is a technician shortage, so staffing is almost never par. In the medical surgical unit, a nurse was checking if a patient was appropriate for the unit. They explained that, usually, the charge nurse would be responsible for this task, but the charge nurse had their own assignments due to staffing. |
|  | The healthcare system inefficiencies may frustrate staff and impact their ability to provide unbiased, actionable feedback for technology innovation. | Frustration with the healthcare system may overshadow or overvalue technology performance. Staff may perceive technology as a temporary solution or another process to implement instead of an actual fix if the root cause is not addressed. Staff may perceive technology as essential if it addresses a pain point of interest even if it does not progress the actual solution to the issue. Additionally, frustration with the healthcare system may overwhelm staff and may cause them to perceive change as too difficult to pursue. | In the ED, a nurse educator was discussing a lack of backwards compatibility between different models of a patient monitor and brand incompatibility when using a newly acquired defibrillator from a different company. They describe how what once were tiny, manageable issues becomes problematic, system-level issues. When discussing the defibrillator’s performance, nurse input heavily focused on what the device was lacking and the workflow challenges they anticipate. In another instance, a nurse educator compared the hospital system to a space station that had surpassed its operational life, where repairs become the new standard. |
|  | Alarm/icon fatigue and desensitization is a prevalent issue. | Technology can contribute towards alarm fatigue if error messages and alarms are not used purposefully or if each device is isolated and alarms independently. | In the ED, a nurse educator was discussing distractions and interruptions. Noting that not all interruptions are bad, these incidents are not tracked. They also mentioned how alarm fatigue, a related and well-documented issue in nursing, is prevalent but not being worked on due to a lack of bandwidth. In the medical-surgical unit, a nurse displayed delayed reactions to alarms. In one instance, the nurse did not react to proceeding bed alarms while charting at the nurse station after disrupting this task by checking on a false alarm. In another instance, the nurse delayed their reaction to a bed alarm while charting outside of a patient room. A patient with a lower-limb amputation was attempting to leave their bed without their prosthetic or mobile assistance. The patient was able to sit upright and position their lower body over the bed before the nurse stopped their current task, assessed the situation, and intervened. |
|  | Drug diversion and theft occur. | Special considerations are required with drug dispensing technology.  Technology should mitigate risks of such behavior with respect for staff, avoiding assumptions of wrongdoing while minimizing workflow disruptions and acknowledging the added burden of such procedures. | In the ED, the medication room is locked and located in a glass room. The glass room was purposeful, as it helps increase visibility, and card access is required to prevent unauthorized access to combat diversion.  In the medical-surgical unit, a nurse was tasked with auditing the automated dispensing cabinet by manually counting each medication. The task was not received well by this particular nurse, voicing that this task was nerve-inducing. On a related note, a different nurse explained they open most medications in the hallway except for narcotics. When opening narcotics, they chose a specific workflow where the medication is opened at the bedside in front of patients for transparency. |
|  | Staff often do not restock supplies and return equipment. | Missing supplies and equipment make task planning difficult for autonomous systems. The technology may also not be returned to its appropriate location. Thus, it is beneficial to be compatible with existing tracking and locating systems or to implement such capabilities directly. | In the PACU, nurses were observed asking for or taking supplies from other nurses, such as gauze, gel, pillows, belonging bags, and IV smart pumps. Additionally, nurses were observed missing items from the miniature supply carts that are outside of each patient pod. In the medical surgical unit, a nurse had difficulty locating a soft call bell. In an attempt to locate the item, the nurse inspected the supply closet, asked different staff members in their unit for the location, and called other units. Eventually, the call bell was returned to the supply closet and located by the unit’s nurse educator. In the ED, a nurse was explaining that the unit had transition from a large, centralized supply system to smaller, decentralized system. When asked about implementing personal carts with their most frequently used supplies, the nurse hypothesized that it would not improve workflow due to having no one to restock these personal carts. |
|  | Multiple staff members compete for the use of limited technology, “ownership” and responsibilities for shared technologies are often unclear. Some resources are shared across units, increasing difficulties locating them. | Lack of supplies and equipment make task planning difficult for autonomous systems. Any newly introduced technology may exacerbate equitable access issues if the appropriate number of units are not accessible to everyone who needs it. | In the PACU, a nurse was preparing for a patient admission. They went to the main nurse station to grab a bladder scanner and then briefly looked for an IV smart pump. After being unable to locate one, the nurses took an IV smart pump from the adjacent patient pod.  In the ED, the unit spent tens of thousands of dollars tethering cables to the corresponding medical device to improve cable management. The solution did not last long; after ~14 days, many tethers were cut by surgical shears. The nurse educator explained that nurses prioritize the patient even if it is at the expense of other nurses, and questioned what was being done after the chaos subsides to rectify. For the same medical device, clinical engineering staff made a plastic anchoring device so a specific cable does not get lost. Even with the presence of the anchoring deice and a label that reads “DO NOT REMOVE”, the cable still goes missing. |
|  | Frontline staff often lack sufficient knowledge of how to troubleshoot/fix equipment. | Device manual design and quick reference support documentation are important. Non-expert users should be able to navigate documentation easily and quickly. Intuitive usage and design are important as staff may not be properly trained to use the device and may be relying on their internal mental model, or the manual may not be accessible in a timely manner. New technical support positions or clinical engineering roles may be needed in the organization as to not overburden nurses with additional non-nursing tasks (fixing and maintaining equipment). | In the PACU, a nurse was observed troubleshooting the interpreting tablet. There appeared to be an issue with touch screen responsiveness. The nurse tapped the screen and inspected the front and back side of the tablet repeatedly and was unable to progress based on the tablet’s feedback. |
|  | Staff have differing personal habits and preferences for technology usage. | At times, these personal preferences contrast with more advanced technology solutions. | In all units, nurses complemented EHR usage with paper notetaking; methods for paper notetaking were nurse and purpose dependent. In the medical-surgical unit, one nurse explained that they were making a task list and reviewing their patients. The task list was on a plain piece of printer paper folded in half that was divided into 6 sections. The nurse wrote one- or two-word phrases with a checkbox for each patient, Later in the shift, the nurse was  creating a medication schedule on paper by transcribing relevant information from the EHR. |
|  | Demographics of frontline staff matter. | Identifying technology innovation requires many different prospectives. | A nurse educator was discussing how experienced nurses are a resource. Experienced frontline staff are knowledgeable but are in higher stress positions and thus lack bandwidth for additional roles Additionally, they may have a hard time identifying tacit tasks and issues. Less experienced staff may have more bandwidth to innovate but lack the appropriate language or knowledge to communicate avenues for innovation that has organizational level impact. |
|  | Staff may have reluctance to adopt new technology. | Intuitive design is important to minimize the barrier to entry of any technology adoption. User interface and user experience are important factors in device design. If improving existing technology, valuable design insights regarding these factors can be made through usability testing of commonly used devices. | There is prevalent usage of paper across all units despite having an electronic documentation system. In the PACU, a nurse preferred to print documentation with relevant information. They clarified that the unit does have complete electronic charting, and that not every nurse does this. They further explained that paper facilitates hand-offs and workflow. In the same unit, a charge nurse was performing a task that using a dry erase marker and a laminated document depicting the unit’s layout. Unrelated to charting, in the ICU, patient transfers were done using transfer sheets and boards despite the presence of ceiling and mobile lifting devices. |
|  | Staff must balance quick, convenient workflows with cost and waste. | Due to sterility concerns, supplies that leave a clean supply room to be used or stored elsewhere (e.g., patient room, supply cart) cannot be returned. Staff must minimize supplies that are removed from the clean supply room and will underestimate supplies when preparing. | In the PACU, preparing for incoming new patients is facilitated by a checklist. Part of the checklist involves supplies to stock in the patient’s room (e.g., urine management, bandages, hygiene). A patient care assistant explained that when staging items in the patient’s room, they intentionally stock less to prevent waste. At the expense of time, the patient care technician stated that they can always get more supplies later. In a different instance, a PCA was staging items on patient beds to assist with patient admissions. They noted that having enough supplies is a limitation; to be efficient, they must work with what is available and minimize the time to get new supplies. The PCA emphasized the importance of timely admissions preparation; a delay in patient admittance delays the entire care team. |
|  | Staff access to nutrition during working hours | Physical resources, such as energy or strength, emotional affect, and physiological responses may be affected by missing regular meals and snacks. User may be at suboptimal mental and health conditions when operating technology so energy expenditure and task complexity to use technology is an important factor. | In the ED, a nurse was preparing to end their shift. Their shift was hectic, and the nurse had no time to eat. They stated that even if they had been offered a food break, it would have just put them behind schedule. In the medical surgical unit, a nurse that was supposed to eat breakfast never had the chance for a break. The nurse mentioned that while they do have food ambassadors for patients, nurses are not guaranteed food or have many chances to eat. |
|  | Staff workflow and prior knowledge sometimes conflict with technology’s pacing and error messages. | Task and/or workflow analysis may help elucidate proper pacing to better fit staff workflow. Allowing users to control specific parameters may help reduce the perception of incorrect error messages, but ethical considerations apply in the event of a user error. User-specific customization may also require additional support staff for these systems | In the PACU, a nurse received two error messages while in the supply room using the automated dispensing cabinet. First, the device issued a verbal notification related to task speed when the nurse grabbed an item. The nurse disagreed, told the machine they did not go too fast, and continued their task. Then, the machine issued an alarm. Unphased, the nurse assumed it was due to a door not being closed properly and finished their task. |
|  | Nurses are often directly or indirectly involved in emotional events that are unrelated to technology yet impact their perception of and their performance with technology. | Emotional events affect staff responses to other work system elements and vary between individuals. | In the ED, a nurse was frustrated with their role. They were tasked with patient coordination, which involved: giving report, reading the patient chart, calling the bed board, and moving the patient around until rooms are available. The frustration was caused by miscommunication, where the nurse is told there is no bed available when there is a bed available for the patient. The nurse perceived the task as labor intensive, a waste of time, and a waste of resources. In the medical-surgical unit, a nurse was observed transcribing patient information from the electronic health record to a piece of paper. The charge nurse explained that the nurse was a bit upset. The transfer the nurse prepared for did not end up coming to the unit; they were now receiving a different patient and had to prepare all over again. |
|  | Nurses express feeling the pressure of being liable for failures that are not within their control. | Incorporating technology without appropriate support may exacerbate this. New technical support roles may be needed in the organization as to not overburden nurses with additional non-nursing tasks. | In the ED, a nurse was contemplating factors that slow down their workflow. One frustration they voiced was the need to be smarter than other healthcare workers, specifically the doctor or pharmacist. They noted that if someone approves an order or procedure that is wrong, the nurse would be perceived as at fault. |
|  | Computer mouse clicks and button presses are needed to use devices and navigate information systems. | User interface, user experience, and human-computer interactions are important factors in device design. Number of clicks and button presses need to be considered. | Nurses expressed that the number of clicks needed to navigate the EHR is an issue. In the ED, a nurse lead was using the EHR. The nurse needed to click the mouse or keyboard just to navigate between over four different windows to acquire and enter information. While navigating the user interface, the nurse stated this task is draining and time consuming. |
|  | Emotional and physical resources are required for equipment usage and maintenance that can be taxing on staff. | Consider minimizing the amount of user input, required feedback that needs human intervention, and tasks that require coordination with nursing staff. New technical support positions or collaborations with clinical engineering may be needed in the organization as to not overburden nurses with additional non-nursing tasks (fixing and maintaining equipment). | In the medical-surgical unit, a nurse was transcribing information from the EHR to a piece of paper. The nurse stated that a lot of mental work needed to prepare and organize medication schedule. A different nurse in this unit was tasked with automated dispensing cabinet medication narcotic and scheduled drug auditing. When retrieving accessing items from the automated dispensing cabinet, nonergonomic postures and repetitive bending were observed. When auditing, the nurse was aggravated and was vocalizing their frustrations. The manual counting made the nurse nervous, and they preferred if they were grouped into batches to make facilitate counting. In the ICU, a staff nurse had a different perspective regarding medication audits. While the task was perceived as easy, the main resource being used is time. |
| Environment (Physical, Socio-organizational, external) | Environments are constantly changing and dynamic. | Technology must be appropriate for constantly changing, dynamic environments. Special considerations are required for mobile and autonomous systems. | In the ICU, a phlebotomy cart appeared outside of a room that was not there originally. A nurse was occasionally grabbing supplies from it. In the PACU, a nurse needed an allergy bracelet. Typically, there is a supply cart adjacent to the nurse station, but it was missing. The nurse, looking for validation, asked to confirm that there had been a supply cart previously. A different nurse at the nurse station replied that a technician took the cart to restock the contents. |
|  | Environments are distracting and overstimulating. | Technology can contribute to sensory overload. | In the medical-surgical unit, a nurse was at the nurse station charting on a desktop computer. Meanwhile, a bed alarm was sounding, LED lights of a call light were blinking red, the keyboard button strokes and mouse clicks were audible, conversations were occurring, and a rapid succession of beeping and pauses was produced by a centralized monitoring system. |
|  | Alarms are prevalent and often. | Staff may struggle to distinguish between multiple competing alarms across different patients. The volume and tone of alarms may not correlate to their urgency. | Alarms were consistently present across all units. Centralized patient monitors constantly beep at the nurse station. Bed alarms sound for various reasons, including when a patient attempts to leave. Patient devices frequently alarm and alert staff members, such as when an IV smart pump has an upstream occlusion or has finished transfusion. The pneumatic tube system beeps with each button press and alerts users when a tube has arrived until someone intervenes. Devices unknown to the care team beep, such as when a foreign external ventricular drain alerted occasionally and caused confusion. |
|  | There is a need to balance accessibility, privacy, and visibility of patients. | Remote abilities may not be feasible due to this balance and some tasks may not be eligible for technology to perform given their interaction with private user data or information. | In the ICU, one nurse described what they’ve deemed the dark side of the unit. In contrast to the rest of the unit, where the patient rooms have more space and are located near the nurses’ station, the dark side is not ideal for critical patients. While being further from the nurses’ station grants patients more privacy, the distance interferes with patient visualization and monitoring. This sentiment was reiterated in an unrelated conversation centered around features of medical devices nurses would like to implement. One nurse recalled using an IV smart pump in an MRI room that could transmit readings into the control room. They suggested implementing remote control capabilities in IV smart pumps for patients with precautions to mitigate the need for PPE every time one enters the room and to save time. They were interested in the ability to purge lines and clear occlusions. Later in the conversation, another nurse described how some patient monitors can alert nurses of errors through sending information to a nurse call device. This nurse did not suggest implementing this capability in the ICU, as remote capabilities may prevent patient visualization and assessment. |
|  | Guidelines from external regulatory bodies and need for HIPAA compliance. | Innovative ideas may not be feasibly implemented due to the need to meet external regulations and ensure patient privacy. Consulting staff knowledgeable on regulatory requirements is critical. | In the PACU, workflow was recently altered to adhere to an external regulatory body. Previously, IV start kits were available near the nurse station. These kits were created by packing necessary supplies in an emesis bin, including: IV fluids, syringes, needles, tape, dressings, labels, tourniquets, alcohol pads, and gauze. Then, nurses would take an IV start kit at their convenience when caring for their patients. After a recent JCAHO visit, the IV start kits were removed. The presence of unlocked sharps created a safety issues, and the presence of adhesive supplies created a sanitary concerns. |
|  | Policies and culture between units are inconsistent. | Generalization is difficult as technology that may be positively received in one unit may not be reciprocated in other units even within the same organization. | In the PACU, a nurse was using phone calls as a primary communication method. When asked about communicating with a phone as opposed to their call device, the nurse stated that the call device is not used consistently in this unit. They contemplated that the lack of usage is possibly due to people not being used to it or just not liking it. The topic came up again when the call device did not work as intended. When attempting to call someone from the device, the nurse had to repeat the extension. When needing to make a second call, the nurse stated that they did not think the receiver would pick up their call device and that they were going to bypass the device entirely by using the phone. They stated that they are impartial to using this call device; that when it works, it's great but typical issues affect usability such as speech recognition. In contrast, staff in the ED communicated heavily through the EHR and call devices. |
|  | Temperature and lighting between units vary. | Variation in lighting and temperature challenges sensor capabilities and calibration. Special considerations are needed for measurement devices that need stable environments. | Certain locations in the hospital were notable for their temperature differences. For example, the operating room was perceived as cold, while the ED trauma room was hot. In the case of the OR, patients were given thermal caps.  Regarding light, pollution from devices and the hallway were present. In the PACU, patients were given a controllable warming system to combat temperature issues. Each patient pod had a thin LED panel from floor to ceiling emitting light. Each patient has access to privacy curtains that  were sometimes used for light damping |
|  | The layout of hospital units are inconsistent. | Generalization is difficult due to these inconsistencies. Special considerations are required for mobile and autonomous systems. | One unit consisted of a long, narrow hallway with a centralized nursing station; one unit was the largest inpatient unit,  had a pod system that catered to different patients, and did not have centralized supplies; one unit was two floors; one unit was U-shaped with resource-specific supplies |
|  | Workspaces are often small and cluttered. | There is limited space for new technologies including both during operation, but also for storage. While floor and table space are limited, ceiling and aerial applications seem promising. | In the medical-surgical unit, movement in small hallways was further impeded by the presence of people as well as furniture and equipment such as: food tray carts, environmental cleaning supplies, workstation on wheels, IV poles, chairs, diagnostic devices, and stretchers. In ED, a handheld device was getting dropped and damaged due to a lack of tablespace in the trauma and resuscitation room. |
|  | Once built, physical layout is not easily adapted to changing technologies or processes. | Technology and required accessories should be compatible with existing infrastructure. The implementation of technology that requires changes to the physical layout may not be feasible without a robust cost benefit analysis. | In the ED, a newly acquired defibrillator was not compatible with the current mounting infrastructure and required custom-made device attachments to integrate into current nurse workflow. In the ICU, an elevator door closed on a nurse during a transfer due to lack of space. The elevator was not designed to fit a stretcher, IV pole, and the care team. |
|  | Organizational cultures may not be conducive to nurse-led innovation. | Nurses-led ideas for technology usage and innovation need organizational support to be implemented. Support involves listening to feedback, implementing feedback in a timely manner, and ensuring the user requirements are properly translated. Issues arise when cost benefits to the organization are not sufficient, despite the value the technology would add to staff quality of life. Ideas, even if not immediately actionable, should be documented and may benefit from thematic analysis and later implementation. | In the PACU, a nurse was describing their frustration with attempting to improve technology. The nurse had experience at a previous workplace that used an app for communicating with families. Wanting to implement the app at their current workplace, the nurse was told the app costs too much money to purchase but that the organization would develop their own version. Even after attending meetings and providing feedback, the nurse did not hear back regarding the app’s actual development. Eventually, the end-product the organization developed did not retain the nurse’s requirements and did not meet expectations. |
|  | Organizational policies make it difficult to change practices. | Value analysis is needed to implement new technology. This may be difficult in units with lower volume patient populations (e.g. units that include pediatric patients in adult populations) or if the technology of interest competes with a company that supplies both the device, the required consumables, and additional supplies. Additionally, the cost value the technology of interest provides the organization sometimes conflicts with the benefits the technology provides for the end-user. Efficient use (e.g. power, consumables, etc.) may need to be implemented by the designer since that protocol may not be provided by the user. | Nurses recognized the need for hospital buy-in to implement a new device or technology, stating that technology needs to benefit the hospital for the greater good. In the ED, the pediatric nurse educator noted the difficulties they had when acquiring a syringe pump. They explained that their patient population has different needs than the general adult population. Thus, their team has difficulties proving value to the hospital due to this mismatch in needs. In the ICU, three nurses were contemplating technology improvements. One nurse ideated about a wireless sats probe. Inspired, another nurse suggested they implement fitting sats probes to patients when admitted to improve workflow; however, they followed their statement by noting that this improvement is an option but is not feasible due to costing money. |
|  | Equipment/supplies are maintained and stocked by different staff from end-users. | Nursing and other knowledgeable healthcare staff are unable to easily change the layout of their supply closets despite knowing the workflow and use cases for supplies. Changes that are implemented are subject to change due to lack of knowledge, as supply management staff do not typically work with nursing staff to maintain supply closets in a way that’s conducive to nursing workflow. Special considerations are needed for supply closet technology integration and automation. | Frustration with supply closet organization was expressed in all units. In the PACU, a nurse educator was interested in understanding their supply usage. They received an inventory list from the materials department with the goal of identifying the items being stored and potential redundant or vestigial supplies that are no longer needed. The nurse educator described the process as confusing despite working with the materials department. They were frustrated that nurses are not able to move objects around and that supply management does not have the workflow knowledge to stock supplies in an efficient manner. When researchers contacted supply management to inquire about technology improvement suggestions, such as a tablet interface that displays the inventory in real-time, supply management was unsupportive and declined to be involved. |
|  | Supply rooms and carts must balance the need for many rapidly accessible supplies against the need for well-organized supplies that are easy to locate. | Offering too many supplies in a unit stockroom can make the stockroom visually overwhelming and difficult to navigate, while offering too few supplies increases the burdens to nurses accessing needed technology. Technology to assist with real-time supply and resource tracking and retrieval is promising. | Supply rooms contain hundreds of items. In the PACU, one closet designated for pre-op general supplies had over 250 unique objects stored on metal wire racks. Objects are separated using wire dividers and are labeled with a barcode that includes the object’s name. The size of the label’s text and the proximity to other labels make reading challenging. During one instance, a nurse was looking for a thin bandage. The nurse did not utilize the barcode labels and was searching for the bandage using visual recall of the packaging. Because the packaging print was on one side of the bandage only, the nurse did not originally see the bandage when they entered the room and located the item when they attempted to leave the supply closet. In the ICU, a nurse spent a considerable amount of time in the supply closet looking for a stopcock valve. After 10 minutes of unsuccessful searching, the nurse found a different object that used a stopcock that would be sufficient for their use case. While having an increased number of supplies made searching difficult, the presence of an alternative option was beneficial. |
|  | Location of technology is often not conducive to nursing workflow. | Appropriate locations for technology are needed. This may not be feasible without organizational support. | In the medical-surgical unit, multiple locations were often needed to prepare for medication passes and patient care. One automated dispensing cabinet is centrally located in the nurse station and is near the kitchen, while another is located towards the end of the hall surrounded by patient rooms. In one instance, a nurse traveled to four different locations to get the water, linens, and medication necessary for a single patient. In another instance, a nurse walked the length of the unit often, repeatedly traveling between the hallway automated dispensing cabinet and the kitchen. This showcases the importance of locating dispensing cabinets near the kitchen and thus drinking water. In the ED, a patient care technician was contemplating how robotic technology could assist with patient bed transfers. They noted that they were unsure if the unit had a lifting assistance devices and was unsure of where to find it. In the PACU, a nurse had retrieved a device from the sanitation machine. They briefly looked for places that could use the sanitized piece of equipment but ended up walking down the hall to Equipment Alcove and stashing it there. |
|  | Presence of liquid reagents, including biological and/or biohazardous materials, on the floor or other surfaces. | Autonomous or mobile systems may inadvertently spread these hazards during movement. Special considerations are required for these systems as well as cleaning and disinfecting applications. Involving the organization’s infection control staff early in the design cycle will assist with proper cleaning and disinfection requirement analysis. | In the medical surgical unit, the care team was discussing a patient room door entry that was slippery during rounds. The environmental staff had mopped already, but the team hypothesize the need for a different cleaning agent. In the ICU, an environmental staff member was mopping the supply closet floor while a nurse looked for an item. The staff member verbally warned that the floor was wet. |
|  | Scheduled (e.g. shift change, rounding) and unanticipated (e.g. aggressive patient, fire) events occur. | Technology may be disrupted during usage and required to deviate from routine behavior during such events depending on organizational policies. | All units had unanticipated events, anticipated time periods of increased activity, and scheduled events that disrupted the typical workflow. In the medical-surgical unit, rounding occurred in the morning and required nurses to pause patient care. When available, nurses met either individually or in pairs with the team to discuss patient care strategies and updates in the case manager office. In the ICU, rounding occurs in the afternoons and causes an increase in workload due to an influx of orders. To mitigate obstructions during a code, a cart in the hallway had sign that read “move cart to SICU classroom during a code red”. Thus, in the event of fire or smoke, the cart must be relocated. In the ED, traumas and resuscitations unexpectedly occur regularly, Each event requires lead resources, such as pod leads and nurse educators, to be reallocated from the rest of the unit. |
|  | Visual cues act as cognitive aids and must be implemented with purpose. | Improper use of visual cues may conflict with prior knowledge may unintentionally promote unsafe practice. Designing features that represent the correct intent may prevent misuse. | In the ICU, there was a fluid bag hanging off a device. The stat nurse mentioned how this was a bad practice, as people tend to use fluid bags that are hanging if they see it. |
| Tools/Technology | The size of technology causes unique challenges. | Large technology takes up storage space and can impede movement; thus portability may be prioritized. Conversely, small technology may be hard to locate or susceptible to theft; thus, tracking may be prioritized. Ergonomics of the device and use under real operating conditions must be considered. | In the PACU, an x-ray technician arrived to image a patient. The patient was inaccessible as is, and the technician had to rotate the bed so that the x-ray machine could fit in the patient’s room. In the ICU, a nurse and two patient care technicians were transferring a patient to the CT room. The nurse considered this patient a relatively easy transfer but there was still an inadequate amount of room in the elevator, causing the door to close on the nurse. In the medical-surgical unit, there were many instances where equipment and devices physically interfered. During one shift, one dedicated task was to move large equipment in the hallway out of the way. |
|  | Speed of technology usage may be inadequate. | Technology must work quickly and not delay care. Workflow must be investigated prior to implementation if speed cannot be improved to ensure appropriateness and to facilitate integration. | In the medical-surgical unit, a charge nurse’s workflow was impacted by slow technology. The charge nurse was delayed multiple times due to waiting for discharge instructions to print. During one of these instances, they rhetorically asked where their paper was. In the PACU, a nurse was explaining the process of using a sanitation device. The device has a 7 minute sanitation cycle and the nurse typically will start the device and walk away. The clarified further that sometimes you return once the sanitation cycle is complete to finish the task, other times someone else finishes the task. |
|  | Broken or malfunctioning equipment is a common occurrence that impacts workflow. | Technology must be robust during usage conditions and resistant to damage. Considerations for storage should be addressed to avoid damage while idle. | In the medical-surgical unit, a broken blood pressure device disrupted a nurse’s workflow. Instead of using the device, the nurse had to locate the manual. Because the manual was not in the expected location, time was spent looking in the nurses station and asking coworkers until the manual was located. In the ED, a nurse was unsuccessfully attempting to charge a device with a bent charger prong. The nurse noted that the improper fit could be a fire hazard. Later that shift, a charger prong was damaged during a patient transport when the move was initiated prior to disconnecting the device. During a different instance, when a nurse was contemplating what impacts their workflow, they mentioned that equipment never works and is not durable enough |
|  | Technology will come in contact with non-sterile biological and environmental materials. | Technology must be able to be quickly, thoroughly disinfected. Unsealed mechanical and articulating components are discouraged due to sterility capabilities and infection control precautions. | In the ED, a cable management sleeve accessory provided by the medical device company was made of an inadequate material. Once exposed to blood, the sleeve would stain. In the medical-surgical unit during rounds, the team was discussing how the floor was slippery in the entry to a room despite being previously mopped by a janitor. |
|  | Updating technology is costly and difficult. | Update costs should be considered and minimize costs and impact. | In the ED, a trauma room monitor is no longer compatible with the new defibrillator. The unit did not intend on replacing the monitor due to the cost. |
|  | Maintaining technology requires staff time and expertise. | Technology should have a low need for ongoing testing/quality control measures that must be performed by frontline staff. New technical support positions may be needed in the organization as to not overburden nurses with additional non-nursing tasks. | In the ICU, there was a device at the nurse station that had printed a strip of paper previously. When asked what the strip of paper was for, a nurse explained that if an alarm goes off this machine prints the waveform signal. The device happened to be out of paper, and a nurse passing by commented how they did not know the device was out of paper and that they had missed this. The device had a backlog of signals to print, and over 10 minutes later was still observed to be printing. |
|  | Lack of backward compatibility and limited interoperability across manufacturers can hinder technology adoption | Technology and required accessories should be compatible with existing equipment. | In the ED, a newly acquired defibrillator was having brand incompatibility issues and certain aspects of the unit’s workflow are no longer feasible. The blood pressure sensor required for the new defibrillator was not the same brand as the organization’s current contract, and thus previously purchased sensors cannot be used with the new device. The streaming monitors and portable defibrillators are different brands, and nurses can no longer switch between devices using the same cables. |
|  | Incompatible technology may appear compatible with inappropriate technologies if visually similar | Technology that appears too similar or is compatible with dissimilar technologies may cause confusion and create safety risk. | In the ED, the unit had two different monitors from the same company, where one model is commonly used in the hallway and one model is used in the room. The cable from one model fits the port of the other but does not function. A nurse remarked how the visual similarity is a safety issue. Further, due to scarcity of cables, moving a patient from a room to the hallway is more difficult. |
|  | Technology is not standardized across different hospitals | Inconsistencies between technology across hospitals can create delays in care for transferred patients. | In the ICU, a local patient that had an accident overseas was recently transferred back from an international hospital. The patient was transferred with necessary equipment from the international hospital that had compatibility issues with the rest of the unit’s equipment. The device was unfamiliar with most of the care team, aside from an expert stat nurse. Verification was performed visually to determine appropriate device output. During a transfer, the alarm from the device had caused a respiratory nurse to ask a coworker to identify the cause of the alarm. Their coworker was unable to determine the cause of the alarm. |
|  | A single patient may require multiple instances or types of devices for treatment | Technology must accommodate the need for many different simultaneous interventions for a single patient. This may heighten issues caused from transporting, where patient equipment requiring wires and cables restrains movement and prevent patients from ambulating far from equipment mounting locations. | In the ICU, a single patient was often observed requiring different equipment. A patient considered the sickest patient in the unit required over 7 IV smart pumps, IV pole, and a kidney machine. A different patient had over 4 IV smart pumps, a ventilator, and an external ventricular drain. |
|  | Personal protective equipment (PPE) is used by staff. | Technology must be compatible with PPE such as gloves, glasses, masks, and gowns. Such equipment impedes the user’s senses (e.g. tactile, auditory, visual). | In the PACU, a nurse wearing a mask was attempting to use a call device. The mask interfered with audio output, causing the nurse to repeat themselves. |
|  | Technology will be moved and transported. | Issues arise due to poor portability, especially cables and wires, highlighting the need for quick disconnects/connects. Patient equipment requiring wires and cables restrains movement and prevent patients from ambulating far from equipment mounting locations | In the ICU, a patient with multiple IV smart pumps, a ventilator, and an external ventricular drain required transport. Before transporting the patient in their bed, staff detached as much equipment as possible to improve maneuverability. Equipment was unattached and placed on bed with the patient or was left on the IV pole. The care team had trouble fitting on elevator due to the wheels of IV pole in combination with the bed. In the ED, the nurse educator emphasized that cable management is a big issue. When caring for a patient, wires were observed getting in the way of the task and forming tangles. In the PACU, a nurse was observed having wire management issues between a blood pressure cuff and oxygen sensor |
|  | Wi-Fi, RFID, and network connectivity issues occur. | Technology must work offline or staff trained to perform tasks while devices are offline as not to compromise patient care. | At the PACU, the card access system for unlocking and opening doors was down. When card access was required and a visitor or worker arrived, the next available staff member manually opened the door. In the ED, a nurse educator mentioned that the unit’s Wi-Fi was notoriously problematic due to the architecture and materials of the unit. The unit has a ”Downtime Cabinet” stocked with paper documentation (e.g., rating scales, SBAR, discharge instructions, medication orders, consents) for when unit needs to do things on paper. |
|  | Time is needed for data transfer and streaming monitors. | Time needed for data transfer and streaming monitors impacts workflow. | In the ED, the process to stream the defibrillator to the trauma room monitors require pressing the screen multiple times, including for powering on the device, menu navigation, monitor selection, and establishing mobile streaming connection.  Additionally, a nurse educator discussed a prevalent complaint when pulling data from the device where the screen takes an inadequate time to turn on and provide feedback. |
|  | Lack of consistent and equitable initial training, continuing education, and support from the device company. | Accessible, standalone device manual design and additional support documentation are important. Non-expert users should be able to navigate documentation easily and quickly. Intuitive usage and design are important as staff may not be properly trained to use the device and may be relying on their internal mental model, or the manual may not be accessible in a timely manner. New technical support positions or collaborations with clinical engineering may be needed in the organization as to not overburden nurses with additional non-nursing tasks (device training). | In the ICU, nurses were contemplating technology innovation. One nurse noted that some technology ideas are promising but highlighted that there is a lack of education and training. They mentioned that not everyone gets taught. In the medical surgical unit, an Inservice event occurred for new medical device related to urination. A representative for the company was present and gave a demonstration of the device to staff using handheld models of genitalia to show device placement. Staff members were then required to sign a sheet confirming they had been present. |
|  | Information screen visibility is an issue. | Since many devices include fixed displays, screen visibility can be impaired during usage that is exacerbated due to improper fit with existing infrastructure and equipment. | In the ED, a nurse was describing the workflow for transporting a patient that requires a defibrillator. Due to the center of mass of the defibrillator, the angle of the screen when mounted on a stretcher siderail did not allow for easy monitor viewing. Instead, nurses put the device at the foot of the bed during transports. |
|  | Broken or malfunctioning equipment is not intuitive to fix. | Broken or malfunctioning equipment needs to have actionable error messages or alternative backup/manual process. Backup devices and spares are needed. | In the PACU, a nurse needed to use a handheld barcode scanner. The scanner was not acting as expected, confusing the nurse. While troubleshooting, the feedback from the scanner was an increased startup time, flashing green lights, and beeping noises. The nurse explicitly showcased this event as an example of technology being annoying. In the ICU, a patient needed to be transferred to the CT room. When arriving, there was another patient in their bed surrounded by their care team waiting for the CT machine in the hallway. The cause of the workflow disruption was due to scanner downtime. |
| Tasks | Tasks can be repetitive. | Unless technology is automating such tasks, technology usage will also be repetitive and thus ergonomically challenging. | In the medical-surgical unit, medicine administration was observed requiring repetitive tasks throughout different phases of the task. To retrieve the medicine using the automated dispensing cabinet, the nurse must move between interacting with the user interface to retrieving the medication from its designated compartment for each medication. Once retrieved prior to opening the medication packaging, the nurse was required to scan each medication. Then, for each medication, it is removed from its packaging and placed in a container. |
|  | High rates of multi-tasking. | Challenges arise for task planning capabilities; difficult for technology that completes a single task. Single task technologies will crowd work space and complicate workflows. | In the medical surgical unit, a nurse was observed talking with their patient while scanning, opening, and administering medication. Another nurse was observed talking on the phone with a provider while navigating the EHR to find information. |
|  | Staff often perform tasks outside of their traditional role/scope; technology may exacerbate this issue. | Task shifting to technology raises ethical concerns as the relationship between task delegation and user responsibility is defined. The scope of technology’s role and the necessary levels of human intervention must be defined before implantation. Challenges arise for autonomous systems and decision-making capabilities. | *In the ED, a charge nurse had to talk with a PCT that was distressed. The PCT was assigned a new role to assist with workflow and relied on computer usage. The PCT expressed they felt their skills were being wasted and that they were just moving patients along. In the ICU, a stat nurse was assigned a facility-to-facility transport. They were not familiar with the process and had to find someone to do it properly. They expressed that if a task is not done often, proficiency is lost.* |
|  | A high rate of interruptions require staff to pause and accommodate different tasks. | When planning tasks for technology, designated tasks must be flexible in task timing and allow the user to interrupt usage. The system will need to communicate if the task can be resumed or must be restarted. | In the PACU, an Xray technician arrived with a machine while a nurse was preparing for medication administration. The nurse stopped their task and started working on the computer. The nurse continued with medication administration as soon as the Xray technician concluded their task. In the ED, a nurse educator discussed being faced with distractions and being displaced from their original goal when walking from one side of the unit to the other. |
|  | Tasks are performed inconsistently by different staff. | Technology development that leverages human data (e.g. learning from demonstration, motion capture, task analysis) will be dependent on the subject chosen for data collection. | *Medication preparation was observed occurring in multiple locations, including the automated dispensing cabinet room, portable computers in the hallway, bedside tables in patient rooms. In the ED, staff were having issues with documenting personal belongings. The issue was different each time, making identifying appropriate training difficult.* |
|  | Tasks can be redundant and unnecessary. | It may be difficult to determine redundant versus necessary, repetitive tasks without expert input. Thus, task and/or workflow analysis are important. | In the medical surgical unit, the team manually transcribes patient care metrics onto a whiteboard during rounds. Some of the information is later put into the computer, but some are vestigial metric related to a previous teletracking system. |
|  | Tasks are completed based on nurses’ assessment of priority, and many are left undone if not deemed as important. | Nurses may not use technology that does not assist with a high priority task. Identifying task priority prior to technology development is important, as is investigating the relationship between frequency of use and cost benefits. | In the ED, a PCT reiterated this sentiment, stating that when understaffed, they will prioritize tasks based on patient hierarchy of needs. In the medical-surgical unit, a patient was requesting a bath while standing in the hallway. The first nurse to talk to the patient, N1, left mid conversation, increasing the patient’s agitation. While the patient was yelling after N1 to help them and talking with another nurse, N2, the charge nurse intervened and had a long discussion with the patient. |
|  | Some tasks are done to provide empathy, comfort, and convenience. | Actions to provide empathy, comfort, and convenience are hard to observe and to implement. Concerns relating to human connection arise. The value of completion by a person with regards to human attributes should be considered for its contribution to the healing environment. | In the PACU, a nurse had lowered the patient’s bed when their family member had arrived so they could be eye-to-eye during their visit. A different nurse chose to use a specific sized needle compatible for two different applications for their diabetic patient to avoid the need for two separate IV insertions. In the medical-surgical unit, multiple members of the care team were discussing the logistics of how to fulfill their patient’s request for access to fresh air. A different nurse on that unit was observed using linen as bed rail coverings / padding for their patient. |
|  | Conflicting information occurs. | Conflicting information challenge decision making processes and autonomous systems. | In the medical-surgical unit, a nurse was discussing with the nurse educator how to proceed with a conflicting order from a medical provider. A doctor had ordered a discharge against medical advice (AMA). The nurse educator explained how discharge instructions are not given if AMA. |
|  | “Gray areas” exist where there is not always a protocol in place. | Subjectivity challenges decision making processes, autonomous systems, and ethics. Systems need to recognize when operating under too much uncertainty and provide fail safes for human or other interventions. | In the ED, a nurse provided an anecdote relating to an explosion in a metal plant. The team had called EMS to ask if patients were decontaminated and EMS provided an ambiguous answer. While the team needed to know this information, ensuring the truth was not possible and the team had to devise care plans using their best judgment. The incident highlighted the need for a disaster protocol, which was created afterwards. |
|  | Patient prioritization when transporting and moving within the hospital is important. | To distinguish between patients and staff, technology will need role identification capabilities with proper responses. This requirement raises ethical and data privacy concerns. | In the ICU, a transport occurred where a nurse and two PCAs moved the patient in their bed from their room to the CT scan room. The nurse was at the foot of the bed, pulling. PCA1 was at the front of the bed, pushing. PCA2 was on the side, supporting. The nurse explained that people always give priority in the hallways and elevators to patients and will hide out of the way during patient transports. |
|  | Documentation is a crucial task. Current technology is not user-friendly and impacts workflow. | The electronic health record and other important documentation software would benefit from incorporating ergonomic design to improve user interface and user experience. | In the ICU charting frequencies are high and the phenomenon of ICU delirium occurs. In the PACU a nurse mentioned having to back chart due to being too busy. In the ED, numerous forms are involved, including: initial chief complaints, initial assessment, various specific assessments (e.g., skin, Glasgow Coma Scale, biophysical, psychosis, and more), patient belongings, ambulance and EMS handover, etc.,. ED nurses expressed that charting, logging, and scanning slows down their workflow; how documentation is mentally draining and time consuming, yet a task that is emphasized.  In the medical-surgical unit, where nurse-patient ratios were high, nurses made their own medication administration organization tools on paper. |
|  | Technology might shape/bias nursing work processes and influence the tasks that nurses deem to be most pressing. | Human interaction and workflow must be considered when assigning tasks for technology. The amount of trust in a technology will impact workflow. | In the ED, a PCT reiterated this sentiment, stating that when understaffed, they will prioritize tasks based on patient hierarchy of needs. In the medical-surgical unit, a patient was requesting a bath while standing in the hallway. The first nurse to talk to the patient, N1, left mid conversation, increasing the patient’s agitation. While the patient was yelling after N1 to help them and talking with another nurse, N2, the charge nurse intervened and had a long discussion with the patient. |
|  | Multimodal (e.g. verbal and visual, paper and electronic) communication is used. | The need to comprehend different methods of communication impact perception and reasoning requirements of technology. | In the PACU, when discussing the patient’s medication, the patient did not know the names or type of their medication. Instead, they gave their nurse a plastic bag filled with their medications. In the ICU supply closet, a nurse (N1) entered and started searching for an item. Another nurse (N2) went to assist, asking what they were looking for. N1 stated they were looking for the rest of this, and proceeded to show N2 a small package that was in their hand. |
|  | There are many tacit and nuanced actions performed by nurses while doing tasks. | Task and/or workflow analysis may help elucidate these actions and promote proper technology design. | In the PACU, a nurse stated how they will perform the IV insertion portion of the patient’s care prior to admitting the patient’s visitors as a courtesy. This workflow was chosen to minimize visitor discomfort. |
|  | Many dexterous tasks occur and involve more than one hand. | The dexterity required to perform tasks challenges the capabilities of manipulation systems. For technology improvements, consider the number of hands and dexterity needed to assemble and use the device and accessories. | In the PACU, IV starts require many bimanual actions, including: don gloves; apply a tourniquet; assemble syringe and needle; open gauze; open swab; swab patient; check patient anatomy; air dry swab area by waving hands; insert needle; remove tourniquet |
|  | Situational awareness is important for safety. Technology may impede this. | Human interaction and workflow must be considered when assigning tasks for technology. Nurses need to be quickly aware what a technology is doing and operational status. | In the ED, a nurse shared an anecdote involving a Code Silver (active shooter on premises). A nurse attended to a gunshot wound patient who had been dropped off in the parking lot while the active shooter was still there, despite having surveillance technology in that area. |
|  | Medications and medical supplies have different material properties that affect behavior (e.g. viscosity and fluid dynamics). | Special considerations are required for dispensing and other fluid flow applications. | In the medical-surgical unit, while prepping a Mini-Bag medication system, a nurse stated that certain medication are notorious for taking a long time to diffuse. |
